# SELENBP1 expression in the prefrontal cortex of subjects with schizophrenia

**DOI:** 10.1038/tp.2015.108

**Published:** 2015-08-04

**Authors:** M Udawela, T T Money, J Neo, M S Seo, E Scarr, B Dean, I P Everall

**Affiliations:** 1 The Florey Institute of Neuroscience and Mental Health, Howard Florey Laboratories, The University of Melbourne, Parkville, VIC, Australia; 2 CRC for Mental Health, Carlton South, VIC, Australia; 3 Department of Psychiatry, The University of Melbourne, Parkville, VIC, Australia; 4 North West Mental Health, Royal Melbourne Hospital, Parkville, VIC, Australia

## Abstract

Selenium binding protein 1 (*SELENBP1*) messenger RNA (mRNA) has previously been shown to be upregulated in the brain and blood from subjects with schizophrenia. We aimed to validate these findings in a new cohort using real-time PCR in Brodmann's Area (BA) 9, and to determine the disease specificity of increased *SELENBP1* expression by measuring *SELENBP1* mRNA in subjects with major depressive disorder and bipolar disorder. We then extended the study to include other cortical regions such as BA8 and BA44. *SELENBP1* mRNA was higher in BA9 (*P*=0.001), BA8 (*P*=0.003) and BA44 (*P*=0.0007) from subjects with schizophrenia. Conversely, in affective disorders, there was no significant difference in *SELENBP1* mRNA in BA9 (*P*=0.67), suggesting that the upregulation may be diagnosis specific. Measurement of SELENBP1 protein levels showed that changes in mRNA did not translate to changes in protein. In addition, chronic treatment of rats with antipsychotics did not significantly affect the expression of *Selenbp1* in the cortex (*P*=0.24). Our data show that elevated SELENBP1 transcript expression is widespread throughout the prefrontal cortex in schizophrenia, and confirm that this change is a consistent feature of schizophrenia and not a simple drug effect.

## Introduction

Levels of selenium (Se) binding protein 1 (*SELENBP1*) expression have been shown to be higher in the dorsolateral prefrontal cortex from subjects with schizophrenia,^1, 2^ with the second study also showing higher levels of *SELENBP1* messenger RNA (mRNA) in the dorsolateral prefrontal cortex from subjects with bipolar disorder (BP) who had psychosis,^2^ leading the authors to suggest that increased *SELENBP1* expression may be associated with a psychotic state rather than a diagnostic criterion. Although the role of SELENBP1 in the central nervous system (CNS) remains largely unknown, there is evidence to suggest it may be involved in neurite growth and remodelling.^3^ Given that dendritic and synaptic proteins are altered in BP and schizophrenia,^4, 5, 6, 7, 8^ SELENBP1 may have a role in the aetiology of these disorders.

To challenge this hypothesis, we investigated whether *SELENBP1* is differentially expressed in the cortex from subjects with schizophrenia, BP or major depressive disorder (MDD). We also addressed the extent of changes in cortical *SELENBP1* expression by measuring mRNA levels in multiple cortical regions, determined whether a two-marker haplotype of *SELENBP1,* previously shown to be nominally associated with risk for schizophrenia (rs10788804, allele A; and rs2800953, allele A or G),^9^ influenced brain *SELENBP1* expression, and whether changes in *SELENBP1* mRNA translated to changes in protein levels. Finally, we determined whether CNS *Selenbp1* expression was altered by chronic exposure to antipsychotic drugs to determine whether changes in the expression of that gene could be involved in their mechanism of action.

## Materials and methods

### Human post-mortem tissue collection

Consent for this study was obtained from the Ethics Committee of the Victorian Institute of Forensic Medicine and the Mental Health Research and Ethics Committee of Melbourne Health. All tissues were obtained from the Victorian branch of the Australian Brain Bank Network held at the Florey Institute of Neuroscience and Mental Health. Psychiatric diagnoses were made according to Diagnostic and Statistical Manual of Mental Disorders, Fourth Edition criteria^10^ by consensus between two senior psychiatrists and a psychologist after an extensive case history review using the Diagnostic Instrument for Brain Studies.^11, 12^ Informed consent for each tissue collection was obtained from the donor or senior next of kin. All subjects were coded to remove subject identities.

Total RNA and protein was obtained from tissue excised from Brodmann's Area (BA) 9 (lateral surface of the frontal lobe, including the middle frontal gyrus superior to the inferior frontal sulcus), BA8 (primarily the superior frontal gyrus extending from the cingulate sulcus on the medial surface to the middle frontal gyrus laterally, bounded caudally by the agranular frontal region (BA6) and ventrally by the granular frontal region (BA9)) and BA44 (opercular region of the inferior frontal gyrus, bounded rostrally by the ascending limb of the lateral sulcus and caudally by the inferior pre-central sulcus), according to Brodmann's criteria, in a cohort of 30 subjects with schizophrenia and 30 control subjects with no history of psychiatric illness, matched closely for age, sex, post-mortem interval and brain pH (schizophrenia cohort; Table 1). Total RNA was also extracted from BA9 from a further 10 subjects with MDD, 10 subjects with BP and 9 control subjects (affective disorder cohort; Table 1). These control subjects were different from those in the schizophrenia cohort in order to match closely for age, sex and brain pH (Table 1). Our previous studies have shown that cohorts of these sizes can detect significant differences of 10% or 20%, respectively, in the mean value of experimental variables.^13, 14^ For genotyping, DNA was extracted from cerebellum tissue taken from the same 30 schizophrenia and 30 control subjects as the schizophrenia cohort. Demographic data of all individuals are provided in Supplementary Table 1. Investigators were kept blinded to diagnosis allocations during sample preparation and subsequent experimentation.

### Antipsychotic-treated rat tissue

Tissue was utilised from male Sprague–Dawley rats treated for a previous study.^15^ Ethics approval was obtained from the University of Melbourne Animal Ethics committee under the Animal Research Act 1985. Briefly, 6-week-old male rats (initially100–150 g), obtained from the breeding colony at Melbourne University, were randomly assigned to treatment groups and treated for a period of 12 months with either vehicle (H_2_O), 1.0 mg kg^−1^ per day haloperidol, 10 mg kg^−1^ per day chlorpromazine or 10 mg kg^−1^ per day thioridazine in the drinking water, a well-established method for long-term delivery of antipsychotics,^16, 17, 18, 19, 20^ in groups of five, with *ad libitum* access to food and water, and maintained at 19±4 °C on a 12-h light/dark cycle. The volume of water consumed was monitored daily to ensure adequate dosing and was renewed every 2 days to minimise any effects of drug degradation. We have previously shown that animal cohorts of this size allow significant differences in CNS expression of 11% or greater to be readily identified.^15^ These drugs were chosen as they were the most commonly recorded antipsychotics used by the subjects in our schizophrenia cohort, and doses were chosen based on previous studies to approximate clinically comparable dopamine D_2_ receptor occupancy.^21, 22, 23^ This route of drug administration is more similar to the most common way of delivering the drugs in the clinical situation; it has the added benefit of causing little stress to the animals. At the completion of the treatment period, all animals received drug-free drinking water for 72 h before being culled. The brains were rapidly removed, frozen in isopentane (Sigma-Aldrich, St Louis, MO, USA) on dry ice and stored at –70 °C. Brains were coded to render the investigator blind to grouping during subsequent testing. For measuring *Selenbp1* mRNA, total RNA was extracted from a tissue section ~3mm thick excised from the right hemisphere immediately posterior to bregma.^24^

### RNA extraction and first-strand complementary DNA synthesis

Total RNA was isolated from ~100 mg frozen tissue samples using 1.0 ml TRIzol reagent (Life Technologies, Carlsbad, CA, USA) according to the manufacturer's instructions. The RNA was treated with DNase I (Life Technologies) at 37 °C for 25–30 min, then purified by phenol/chloroform extraction and stored at −80 ºC. RNA quantity and quality were analysed by spectrophotometer readings. DNA contamination was checked by PCR using primers specific for genomic DNA. RNA integrity numbers were determined using an Agilent 2100 bioanalyser (Agilent Technologies, Santa Clara, CA, USA).

First-strand complementary DNA was synthesised from 2 μg RNA using 100 units Moloney Murine Leukemia Virus reverse transcriptase with 2.5 μM random decamers and 2.5 μM oligo dT primers (Life Technologies), 0.5 mM of each dNTP and 20 units RNase inhibitor in 1 × reverse transcriptase buffer (50 mM Tris-HCl (pH 8.3), 75 mM KCl, 3 mM MgCl_2_ and 5 mM dithiothreitol) in a final volume of 20 μl. The reaction was incubated at 44 °C for 1 h then inactivated at 92 °C, and the product was stored at −20 °C.

### Real-time PCR

Complementary DNA was used as a template for real-time PCR performed with SYBR green detection using a Bio-Rad iQ5 Real-Time PCR Detection System (Bio-Rad Laboratories, Hercules CA, USA). Reactions were performed in triplicate in 50 μl volume containing complementary DNA diluted 1:125, 0.4 nM primers and 1 × IQ SYBR green supermix (Bio-Rad Laboratories), with cycling conditions of 95 °C for 3 min, 40 cycles of 30 s each at 95, 57 and 72 °C, followed by a melt curve. Quantitative PCR data were acquired using iQ5 optical system 2.0 software (Bio-Rad Laboratories).

In human tissue, normalised relative quantities of *SELENBP1* mRNA were determined relative to the geometric mean of three reference genes, peptidylprolyl isomerase A (*PPIA*), glyceraldehyde-3-phosphate dehydrogenase (*GAPDH*) and alpha synuclein (*SNCA*), that were chosen from nine genes tested in post-mortem CNS tissue as the most stable by geNorm analysis^25^ (*M*=1.398, 1.386 and 1.407, respectively). In rat tissue, normalised relative quantities of *Selenbp1* mRNA were calculated using reference genes succinate dehydrogenase complex, subunit A, flavoprotein (*Sdha*), mitogen-activated protein kinase kinase 5 (*Map2k5*) and mitogen-activated protein kinase 6 (*Mapk6*). See Supplementary Table 2 for primer sequences. All amplicons were confirmed by sequencing before undertaking quantitative PCR. The relative quantities of the reference genes were not different between the analysis groups, and therefore were suitable reference genes for the present study.

### Genotyping

Approximately 25 mg of frozen CNS tissue was homogenised using the Minilys homogeniser Precellys Minilys homogenizer (Bertin Technologies, Montigny-le-Bretonneux, France) in 80 μl phosphate-buffered saline by agitating twice for 30 s at a velocity of 5000 r.p.m., with incubation on ice for 30 s between agitations. DNA was then extracted using the QIAamp DNA Mini kit (Qiagen, Hildon, Germany) according to the manufacturer's instructions. The quality and concentration of the DNA was assessed by NanoDrop (Thermo Scientific, Waltham, MA, USA).

Samples were genotyped at single-nucleotide polymorphisms (SNPs) rs10788804 and rs2800953 with the Sequenom MassARRAY MALDI-TOF genotyping system (Sequenom, San Diego, CA, USA) using Sequenom iPLEX Gold chemistries, according to manufacturer's instructions. Primer sequences are given in Supplementary Table 2. Data analysis was performed in a semi-automated manner using the Typer 4.0 Analyser Software (Sequenom). All genotype calls not assessed as ‘conservative' by the analysis programme were manually checked and discarded if a clear call could not be made.

### Western blotting

**Figure 1:**
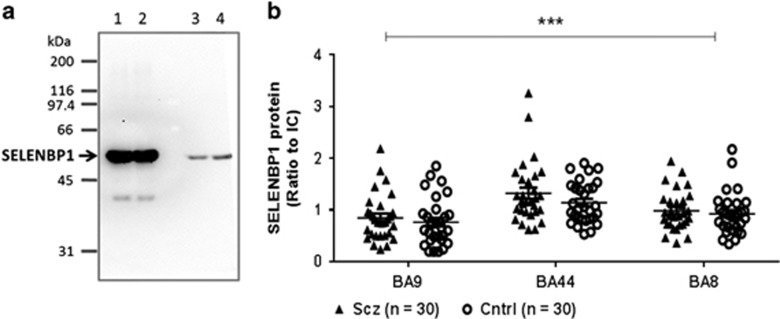
SELENBP1 protein in schizophrenia. (**a**) Western blot of SELENPB1 expression in SH-SY5Y cells transfected with human SELENBP1 (lane 1, 5 μg and lane 2, 10 μg protein) and human central nervous system (CNS) tissue (lane 3, 20 μg and lane 4, 30 μg protein). (**b**) SELENBP1 protein expression in BA9, BA44 and BA8 from subjects with schizophrenia (Scz; ▴) and control (Cntrl; o). Error bars represent mean±s.e.m. ***P<0.001, two-way analysis of variance.

Homogenates were prepared at 5% (w/v) in 10 mM Tris (pH 7.4) containing 1% (w/v) SDS and 1 mM fresh sodium orthovanadate (Sigma-Aldrich), and the protein concentrations were measured using DC Protein Assay (Bio-Rad Laboratories). Twenty-five micrograms total protein of each sample were loaded in duplicate onto a polyacrylamide gel (4% stacking gel and 10% running gel) and separated by electrophoresis (150 V constant), then transferred (100 V constant, 1 h) onto a Hybond-ECL nitrocellulose membrane (GE Health Life Sciences, North Shore, NSW, Australia). Equal protein loading and transfer were confirmed by staining with 0.1% ponceau S in 3% trichloroacetic acid. Nitrocellulose membranes were then blocked in 5% non-fat milk powder/Tris-buffered saline with 0.1% Tween-20 (Sigma-Aldrich) for 1 h at room temperature and then incubated with mouse anti-SELENBP1 antibody (M061-3; MBL International, Woburn, MA, USA) at 4 °C overnight at 1/2000 dilution, followed by goat anti-mouse secondary antibody conjugated to horseradish peroxidase (Dako, Glostrup, Denmark) at 1/2000 for 1 h at room temperature. Antigenic bands were visualised using Pierce Supersignal West Pico chemiluminescent substrate (Thermo Scientific) and the image was captured with a Kodak 440 CF imaging system (Eastman Kodak, Rochester, NY, USA). The antibody specificity was confirmed by the presence of a single band in human CNS tissue homogenate at the known molecular weight of 56 kDa, and overexpression in cell lysate from SH-SY5Y cells transiently transfected with human SELENBP1 (Figure 1a).

To control for inter-blot variation, an internal control membrane sample, prepared from the cerebellum of a human subject that was not part of the cohorts used, was run in 12 wells on two gels to establish intra- and inter-blot variation for SELENBP1 levels.^26^ The internal control was run in duplicate on every gel and gels were imaged so that the optical density of this sample fell within the mean±1 s.d. obtained from the initial two gels. The density of SELENBP1 protein in each sample was then expressed as a ratio to the internal control.

### Statistical analysis

Data from subjects and rats were grouped according to diagnosis or treatment with the identity of the groups kept blind during analyses. Demographic data of the cohorts were analysed by Student's *t*-test or one-way analysis of variance across the diagnostic groups for continuous variables, which were all normally distributed, and *χ*^2^-test for non-continuous variables. Correlations between experimental data and continuous potential confounding factors were determined by Pearson correlation. Owing to the small cohort size, of the relationships that reached significance (*P*<0.05), only those with a *r*^2^ value of more than 0.49 were considered a strong correlation^27^ for further analysis by analysis of covariance, provided the factor was not different between the groups.^28^ Normality of the data distributions were assessed using D'Agostino and Pearson omnibus normality tests for the human data and Kolmogorov-Smirnov normality tests for the rat data. Variance in *SELENPB1* mRNA with diagnosis or treatment was analysed using Student's *t*-test or one-way analysis of variance. These parametric tests were chosen due to their low sensitivity to deviations from normality and low false-positive rates.^29, 30, 31^ To correct for multiple testing in human tissue, Bonferroni corrections were applied, resulting in *P*<0.013, considered statistically significant (analysis performed in schizophrenia cohort in three regions and in affective disorder cohort in one region; *n*=4). Two-way analysis of variance was used to analyse variation in SELENBP1 protein, with diagnosis and region as primary variables, and to assess SNP × mRNA interactions using diagnosis and genotype or allele as primary variables. All analyses were performed using GraphPad Prism version 5.01 (GraphPad Software, San Diego, CA, USA). Values are represented as mean±s.e.m.

## Results

### Demographic data

#### Schizophrenia

There were no significant differences in mean age (*P*=0.91), post-mortem interval (*P*=0.48), RNA integrity number (*P*=0.52 BA9, *P*=0.92 BA44 and *P*=0.74 BA8), brain hemisphere (*χ*^2^_1_=2.07, *P*=0.15) or sex ratio (*χ*^2^_1_=0.0, *P*=1) between subjects with schizophrenia and controls (Table 1). There was a significant difference in the number of subjects that died by suicide (*χ*^2^_1_=9.23, *P*=0.002), thus SELENBP1 data were analysed comparing non-suicide with suicide completers. There was significant variation in brain pH between schizophrenia and control (*P*=0.04), rendering this an irresolvable confound; however, the experimental data showed no strong correlation with pH or any other potential confounds (*r*^2^<0.17; Supplementary Tables 3 and 4), indicating these factors would not affect the outcome.

#### Affective disorders

There were no significant differences in age (F_2,28_=0.11, *P*=0.90), post-mortem interval (F_2,28_=2.47, *P*=0.10), RNA integrity number (F_2,27_=1.40, *P*=0.26) or sex ratio (χ^2^_2_=0.11, *P*=0.94) between groups, and duration of illness was not different between BP and MDD (*P*=0.62; Table 1). There was significant variance in the rate of suicide (*χ*^2^_2_=14.2, *P*<0.001). pH was higher in MDD compared with control (F_2,28_=5.38, *P*=0.01; Table 1); however, it showed no strong correlation with *SELENBP1* mRNA (*r*^2^=0.24; Supplementary Table 3). All other variables also showed no strong correlation with the experimental data (Supplementary Table 3).

### *SELENBP1* mRNA in schizophrenia and affective disorders

#### Schizophrenia

Levels of mRNA for individual reference genes varied between cortical region (F_2,531_=47.63, *P*<0.0001; not shown), and therefore each brain region was analysed individually.

**Figure 2:**
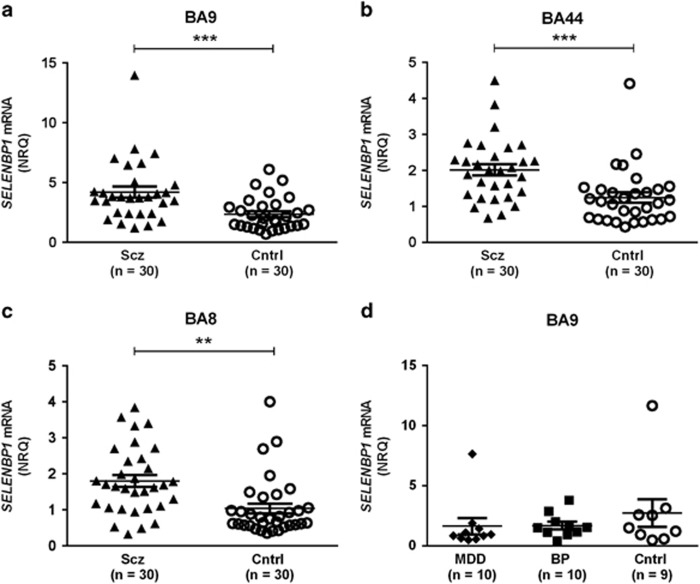
*SELENBP1* messenger RNA (mRNA) in schizophrenia and affective disorders. *SELENBP1* mRNA expression in (**a**) BA9, (**b**) BA44 and (**c**) BA8 from subjects with schizophrenia (Scz; ▴) and control (Cntrl; O), as well as in (**d**) BA9 from subjects with major depressive disorder (MDD; ♦), bipolar disorder (BP; **▪**) and control (□). Error bars represent mean±s.e.m. **P<0.01 and ***P<0.001; *t*-test.

The expression data did not meet normality criteria in the control group in BA9 or BA8 or in the schizophrenia group in BA44. In BA9, *SELENBP1* mRNA was significantly higher in subjects with schizophrenia (4.20±0.47) compared with control (2.36±0.25; *t*_58_=3.47, *P*=0.001; Figure 2a). *SELENBP1* mRNA was also higher in subjects with schizophrenia in BA44 (2.01±0.16 schizophrenia, 1.25±0.15 control; *t*_58_=3.57, *P=*0.0007; Figure 2b) and BA8 (1.77±0.17 schizophrenia, 1.04±0.15 control; *t*_58_=3.15, *P=*0.003; Figure 2c).

Comparing suicide completers with non-suicide subjects showed no significant differences in *SELENBP1* mRNA (BA9 *t*_58_=1.97, *P*=0.053; BA44 *t*_58_=0.17, *P*=0.17; BA8 *t*_58_=0.13, *P*=0.90; Supplementary Figure 1, left); therefore, suicide was unlikely to affect the results. Removing the control subjects and comparing suicide with non-suicide within the diagnosis of schizophrenia also showed no significant difference (BA9 *t*_28_=0.63, *P*=0.53; BA44 *t*_28_=0.12, *P*=0.91; BA8 *t*_28_=1.13, *P*=0.27; Supplementary Figure 1, right).

#### Affective disorders

To determine the diagnostic specificity of mRNA changes observed in schizophrenia, *SELENBP1* mRNA was measured in BA9 from subjects with MDD and BP. Data were not normally distributed in MDD and control. There was no significant variance in *SELENBP1* mRNA with diagnosis (1.64±0.68 MDD, 1.70±0.31 BP, 2.74±1.16 control; F_2,27_=0.55, *P*=0.54; Figure 2d).

*SELENBP1* mRNA was not different between suicide completers and non-suicide subjects, with or without controls included (*t*_27_=0.63, *P*=0.53 and *t*_18_=0.07, *P*=0.95, respectively; data not shown).

### SELENBP1 protein in schizophrenia

The data distribution did not reach normality criteria in the schizophrenia group in BA9 and BA44 or in the control group in BA8. Two-way analysis of variance of SELENBP1 protein showed variance with region (F_2,174_=13.76, *P*<0.0001), but not diagnosis (F_1,174_=2.53, *P*=0.11; Figure 1b), and no interaction between the two variables (F_2,174_=0.30, *P*=0.74).

Comparing suicide completers with non-suicide subjects showed variance with region (F_2,174_=7.98, *P=*0.0006), but not suicide status (F_1,174_=0.01, *P*=0.91), and no interaction between the two variables (F_2,174_=1.98, *P*=0.14; Supplementary Figure 2, top). Upon removal of the control subjects from the analysis, there was still variance with region (F_2,84_=6.50, *P*=0.002) and not suicide status (F_1,84_=0.28, *P*=0.60), and no interaction between the two (F_2,84_=1.77, *P*=0.18; Supplementary Figure 2, bottom).

### Genotype effect on *SELENBP1* expression

Of the two SNPs tested, the primer set for rs2800953 did not allow for the elucidation of genotype in any of the samples, possibly due to inefficient primer binding. Analysis of the data for rs10788804 showed no deviation from Hardy–Weinberg equilibrium (*χ*^2^=0.04, *P*<0.05).^32^ There was no effect of genotype (BA9 F_2,50_=0.32, *P*=0.73; BA44 F_2,50_=2.42, *P*=0.10; BA8 F_2,50_=0.87, *P*=0.43; Table 2) or allele (0.06<*P*<0.78; Supplementary Table 5) on *SELENBP1* mRNA levels.

### *Selenbp1* mRNA in antipsychotic-treated animals

**Figure 3:**
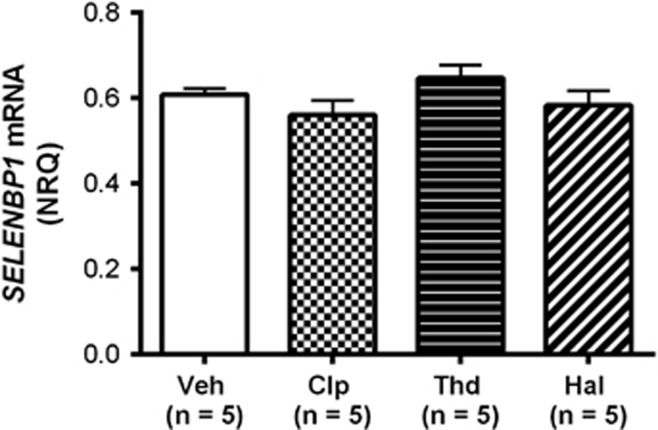
*Selenbp1* messenger RNA (mRNA) in antipsychotic-treated animals. Selenbp1 mRNA expression in central nervous system from rats treated with vehicle (Veh), 10 mg kg^−1^ per day chlorpromazine (Clp), 10 mg kg^−1^ per day thioridazine (Thd) or 1.0 mg kg^−1^ per day haloperidol (Hal). Error bars represent mean±s.e.m.

Data were normally distributed for each treatment group. Antipsychotic drugs had no effect on *Selenbp1* mRNA levels in rats (0.61±0.01 vehicle, 0.56±0.03 chlorpromazine, 0.65±0.03 thioridazine and 0.58±0.03 haloperidol; F_1,19_=1.57, *P*=0.24; Figure 3).

## Discussion

We found *SELENPB1* mRNA to be higher in subjects with schizophrenia in all CNS regions examined, BA8, BA9 and BA44. By contrast, we showed no difference in levels of *SELENBP1* mRNA in BA9 from subjects with MDD or BP compared with control, suggesting that there may be diagnosis selectivity in changes in cortical expression of that gene. Increased *SELENBP1* mRNA levels did not translate to increases in protein levels. Rats treated with the same antipsychotics as those taken by subjects in this cohort showed no differences in CNS levels of *Selenbp1* mRNA, indicating that changes in gene expression in human cortex are unlikely to be simply due to antipsychotic treatment. Finally, we found no association between SNP rs10788804 and *SELENBP1* mRNA levels.

Our findings of higher levels of *SELENBP1* mRNA in the dorsolateral prefrontal cortex of subjects with schizophrenia are in agreement with previous studies,^1, 2^ and we further show that these changes are likely to be widespread throughout the cortex of subjects with the disorder. We were unable to confirm higher *SELENBP1* mRNA levels in BP with psychotic features^2^ owing to small sample size and insufficient case history. Although psychotic state was not well recorded in our subjects with BP, half of the subjects had been prescribed antipsychotics; separating BP subjects based on antipsychotic treatment showed no effect on *SELENBP1* mRNA expression levels (*t*_8_=0.99, *P*=0.35; data not shown). In this regard, of interest is a small study of patients during their first hospitalisation with a schizophrenia spectrum disorder (schizophrenia, schizoaffective disorder or schizophreniform disorder) that reported no difference in levels of *SELENBP1* mRNA in blood compared with controls.^33^ Significantly, that study involved patients in their first admission, whereas other studies utilised patients with an established diagnosis of schizophrenia, suggesting that differences in *SELENBP1* expression may depend on disease state. Overall, our study adds to previous data showing upregulated *SELENBP1* expression in schizophrenia; the consistency of this effect, together with mRNA being shown to be altered in blood as well as brain,^1^ indicates that it could be a candidate for inclusion in a panel of biomarkers that could be used to aid in diagnosis.

Rats treated with the same antipsychotics as those prescribed to subjects in the schizophrenia cohort showed no differences in CNS levels of *Selenbp1* mRNA. Not measuring CNS levels of antipsychotic drugs is a limitation to this study that is common to many studies in rats that use this, and other, delivery methods. Importantly, there are a number of CNS-driven behavioural studies, particularly focussed on modulating dopaminergic activity, that show that the delivery of antipsychotic drugs in the drinking water does result in appropriate levels of antipsychotic drugs in rat CNS.^19, 34, 35, 36, 37^ In our current study, our results are in line with previous studies, and our data show no significant relationship between antipsychotic drug dose and *SELENBP1* mRNA levels in schizophrenia,^1, 2^ thereby indicating that changes in *SELENBP1* gene expression in human cortex is a feature of the disorder rather than an antipsychotic drug effect. Our finding of no association between genotype at rs10788804 and *SELENBP1* mRNA levels suggests that changes in gene sequence at this site do not affect expression levels. However, previous studies showing association of polymorphisms^9^ and copy number variations^38^ in the *SELENBP1* gene with schizophrenia support a role of this gene in the aetiology of the disorder.

Although there was a marked change in *SELENBP1* mRNA expression in the CNS in schizophrenia, we saw no differences in total SELENBP1 protein with diagnosis. This could indicate that there would be no functional outcome from our findings on mRNA. However, it is now recognised that for some genes a neuron must rapidly synthesise protein in response to stimuli,^39^ particularly within specific compartments of the cell such as dendrites.^40^ Thus, the changed expression levels of *SELENBP1* could affect the responsiveness of a neuron if it needs to rapidly increase levels of SELENBP1 protein. Findings from other studies indicate that there are altered SELENBP1 protein levels in subjects with schizophrenia. The difference in outcomes from our study and other studies could be related to the sensitivity of the techniques used to measure SELENBP1. For example, a proteomic study using 2-dimensional difference gel electrophoresis found that SELENBP1 protein levels were lower in post-mortem liver from subjects with schizophrenia, but higher in red blood cells from a separate group of patients,^41^ which is in line with mRNA findings in blood.^1, 2^ By using a similar technique, another study detected higher SELENBP1 protein in the prefrontal cortex from a small number of suicide subjects with no known neurodegenerative disease,^42^ whereas we saw no significant difference with suicide; however, that study used only suicide subjects that had died from hanging, and SELENBP1 is known to be induced under hypoxic conditions.^43^ Previously, increased glial and decreased neuronal immunohistochemical staining of SELENBP1 protein was observed in the dorsolateral prefrontal cortex from three subjects with schizophrenia compared with control.^1^ Although this study has significantly more power than that using immunohistochemistry, western blots do not allow us to examine localised expression; we may not be detecting changes occurring in a cell-type or -compartment-specific manner in this study. Thus, a more detailed examination of SELENBP1 protein in the CNS could reveal a physiological outcome of upregulated mRNA in schizophrenia.

Although the exact function of SELENBP1 has yet to be characterised, it is known to bind Se,^44, 45^ a trace element involved in neuroprotection.^46, 47, 48, 49, 50, 51^ It is of note that Se deficiency has been associated with higher rates of schizophrenia^52, 53^ and some studies have shown lower plasma Se concentrations in patients with schizophrenia,^54, 55^ whereas others showed that Se is unchanged in the blood and serum of patients.^56, 57^ Thus, it would be beneficial to determine the Se concentrations in CNS regions in subjects with schizophrenia to ascertain whether there is a link between Se levels and *SELENBP1* expression. The inverse relationship demonstrated between SELENBP1 expression and cell growth,^58, 59^ and the observed association of SELENBP1 with neuronal cell outgrowth,^3^ suggests that changes in central SELENBP1 could be linked to aberrant cell growth in the brains of people with schizophrenia;^60, 61, 62^ however, more detailed studies are needed to determine its exact function in the CNS and its implications in disease aetiology.

With this study, it has now been shown in three separate cohorts that *SELENBP1* mRNA is upregulated in the frontal cortex of subjects with schizophrenia, an effect not attributed to antipsychotic medication. This supports the notion that SELENBP1 may have a role in the pathophysiology of schizophrenia. These findings warrant further characterisation of SELENBP1 and its role in CNS function.

## Figures and Tables

**Table 1:** Demographic data

	*Schizophrenia cohort*		*Affective disorder cohort*		
	*Scz (*n*=30)*	*Control (*n*=30)*	*MDD (*n*=10)*	*BP (*n*=10)*	*Control (*n*=9)*
Age (years)	48.4±3.0	48.9±3.1	62.4±5.0	59.8±4.0	62.4±4.6
PMI (h)	40.5±2.4	43.2±3.0	39.1±5.0	31.1±4.5	47.4±6.0
Brain pH	6.26±0.03	6.40±0.03	6.58±0.06	6.35±0.06	6.29±0.09
					
RIN BA9	8.81±0.09	8.93±0.16	6.77±0.27	5.71±0.53	6.17±0.51
BA44	6.04±0.29	6.00±23	NA	NA	NA
BA8	7.87±0.16	7.78±0.26	NA	NA	NA
					
Brain hemisphere	30L	28L/2R	10L	10L	10L
Sex	24M/6F	24M/6F	6M/4F	6M/4F	6M/3F
Suicide	8/30	0/30	8/10	3/10	0/9
DOI (years)	21.8±2.8	NA	17.6±3.4	20.3±4.1	NA
Drug dose (mg)^a^	560±96	NA	NA	213±146	NA
Lifetime exposure (mg)^a^	13.3±3.2	NA	NA	0.44±0.35	NA
Anticholinergic use	13/30	NA	0/10	2/10	NA
Benzodiazepine use	12/30	NA	1/10	5/10	NA

Abbreviations: BP, bipolar disorder; DOI, duration of illness (years); F, female; M, male; MDD, major depressive disorder; NA, not applicable; PMI, post-mortem interval (h); RIN, RNA integrity number; Scz, schizophrenia.

a Expressed as chlorpromazine equivalents. Mean±s.e.m.

**Table 2:** SNP analysis at rs10788804

*Genotype*	*SELENBP1 mRNA levels*					
	*BA9*		*BA44*		*BA8*	
	*Scz (*n*=27)*	*Control (*n*=28)*	*Scz (*n*=27)*	*Control (*n*=28)*	*Scz (*n*=27)*	*Control (*n*=28)*
GG	3.87±0.56	2.45±0.43	1.67±0.20	1.21±0.19	1.68±0.35	1.01±0.21
AG	4.57±0.87	2.16±0.43	2.26±0.27	1.06±0.15	1.82±0.23	0.94±0.20
AA	5.13±1.15	2.70±0.68	2.77±0.88	1.77±0.67	2.27±0.54	1.42±0.67

Abbreviations: mRNA, messenger RNA; Scz, schizophrenia; SNP, single-nucleotide polymorphism.

Mean±s.e.m.
