# Supplementary material for: SELENBP1 expression in the prefrontal cortex of subjects with schizophrenia
Source: Transl Psychiatry. 2015 Aug 4;5(8):e615–. doi: 10.1038/tp.2015.108 (PMC4564563; doi:10.1038/tp.2015.108)
Supplement: Supplementary Information [file tp2015108x1.pdf]

Supplementary Table 1: Demographic data of subjects

	Age (years)	PMI (hours)	Brain pH	Hemis-phere	Sex	Suicide	DOI (years)	Cause of death	Drug dose (mg)[a]	lifetime exposure (mg)[a]	Anti-cholinergic use	BZP use	ancestry
Control group 1													
C1	52	22	5.98	left	male	no	N/A	Pulmonary thromboembolism	N/A	N/A	N/A	N/A	CEU
C2	25	50	6.48	left	male	no	N/A	Exsanguination	N/A	N/A	N/A	N/A	CEU
C3	53	44.5	6.56	left	male	no	N/A	Ischaemic Heart Disease	N/A	N/A	N/A	N/A	CEU
C4	68	41	6.06	left	male	no	N/A	Aortic Stenosis	N/A	N/A	N/A	N/A	CEU
C5	43	51	6.43	left	male	no	N/A	Coronary Arterial Atheroma	N/A	N/A	N/A	N/A	CEU
C6	72	39	6.21	left	male	no	N/A	Coronary Arterial Atheroma	N/A	N/A	N/A	N/A	CEU
C7	68	69	6.59	left	male	no	N/A	Coronary Arterial Atheroma	N/A	N/A	N/A	N/A	CEU
C8	68	38	6.32	left	female	no	N/A	Acute Asthma	N/A	N/A	N/A	N/A	CEU
C9	22	51	6.58	left	male	no	N/A	Exsanguination	N/A	N/A	N/A	N/A	CEU
C10	66	43	6.37	left	female	no	N/A	Acute Myocardial Infarction	N/A	N/A	N/A	N/A	CEU
C11	42	63	6.34	left	male	no	N/A	Cardiomegaly	N/A	N/A	N/A	N/A	CHB
C12	50	65	6.4	left	male	no	N/A	Ischaemic Heart Disease	N/A	N/A	N/A	N/A	CEU
C13	42	26	6.32	left	male	no	N/A	Coronary Arterial Atheroma	N/A	N/A	N/A	N/A	CEU
C14	26	24	6.42	left	male	no	N/A	Electrocution	N/A	N/A	N/A	N/A	CEU
C15	39	52	6.26	left	female	no	N/A	Mitral Valve Prolapse	N/A	N/A	N/A	N/A	CEU
C16	25	35	6.15	left	male	no	N/A	Right Ventricular Hypertrophy	N/A	N/A	N/A	N/A	CEU
C17	65	41	6.56	left	male	no	N/A	Ischaemic Heart	N/A	N/A	N/A	N/A	CEU

								Disease					
C18	53	12	6.34	left	male	no	N/A	Pulmonary thromboembolism	N/A	N/A	N/A	N/A	CEU
C19	43	45	6.25	left	male	no	N/A	Drowning	N/A	N/A	N/A	N/A	CEU
C20	26	46.5	6.37	left	male	no	N/A	Electrocution	N/A	N/A	N/A	N/A	CEU
C21	48	56	6.38	right	male	no	N/A	Ischaemic Heart Disease	N/A	N/A	N/A	N/A	CEU
C22	36	60	6.4	left	female	no	N/A	Food Aspiration	N/A	N/A	N/A	N/A	CEU
C23	50	69	6.43	left	male	no	N/A	Ischaemic Heart Disease	N/A	N/A	N/A	N/A	CEU
C24	46	46.5	6.08	left	male	no	N/A	Acute Myocardial Infarction	N/A	N/A	N/A	N/A	CEU
C25	71	50	6.33	right	male	no	N/A	Ischaemic Heart Disease	N/A	N/A	N/A	N/A	CEU
C26	65	20.5	6.47	left	male	no	N/A	Acute Myocardial Infarction	N/A	N/A	N/A	N/A	CEU
C27	73	28	6.37	left	female	no	N/A	Uterine Cancer	N/A	N/A	N/A	N/A	CEU
C28	22	62	6.39	left	male	no	N/A	Iatrogenic Haemorrhage	N/A	N/A	N/A	N/A	CEU
C29	59	20.5	6.58	left	female	no	N/A	Congestive Cardiac Failure	N/A	N/A	N/A	N/A	CEU
C30	48	24	6.37	left	male	no	N/A	Coronary Arterial Atheroma	N/A	N/A	N/A	N/A	CEU
Schizophrenia													
S1	51	20	5.98	left	male	no	32	Ischaemic Heart Disease	2000	64.0	no	yes	CEU
S2	25	49	6.38	left	male	yes	2	Combined Drug Toxicity	200	0.4	no	no	CEU
S3	53	37	5.98	left	male	no	30	Intestinal Ischaemia	1700	51.0	yes	no	CEU
S4	67	21	6.46	left	male	no	36	Pneumonia	75	2.7	yes	no	CEU
S5	44	32	6.28	left	male	no	23	Ischaemic Heart Disease	600	13.8	no	no	CEU

S6	71	48	6.45	left	male	no	53	Food Aspiration	150	8.0	yes	yes	CEU
S7	53	43	6.23	left	male	no	7	Food Aspiration	200	1.4	no	no	CEU
S8	69	44.5	6.38	left	male	no	47	Ischaemic Heart Disease	100	4.7	yes	no	CEU
S9	22	37	6.17	left	male	yes	3	Combined Drug Toxicity	200	0.6	no	no	CEU
S10	65	50	6.35	left	female	no	18	Ruptured Abdominal Aneurysm	550	9.9	yes	yes	CEU
S11	41	31	6.2	left	male	yes	11	Combined Drug Toxicity	500	5.5	no	yes	CEU
S12	42	47	6.26	left	male	no	22	Coronary Arterial Atheroma	1000	22.0	yes	no	CEU
S13	26	52	6.39	left	male	yes	2	CO Poisoning	500	1.0	yes	yes	CEU
S14	47	50	6.31	left	female	no	20	Pneumonia	600	12.0	no	yes	CEU
S15	48	30	6.62	left	male	no	24	Bronchopneumonia	1250	30.0	no	yes	CEU
S16	35	15	6.26	left	female	no	7	Coronary Arterial Thrombosis	300	2.1	yes	no	CEU
S17	23	78	6.19	left	male	yes	5	Multiple Injuries	300	1.5	yes	no	CEU
S18	55	25	6.1	left	male	no	33	Coronary Arterial Atheroma	400	13.2	yes	yes	CEU
S19	66	39.5	6.49	left	male	no	45	Bronchopneumonia	1200	54.0	no	yes	CEU
S20	47	32.5	6.41	left	male	no	27	Ischaemic Heart Disease	530	14.3	no	no	CEU
S21	70	46	5.8	left	male	no	20	Bronchopneumonia	N/D	N/D	no	no	CEU
S22	65	42	6.29	left	male	no	36	Bronchopneumonia	460	16.6	yes	no	CEU
S23	71	36	5.84	left	female	no	48	Chronic Obstructive Airways Disease	N/D	N/D	no	no	CEU
S24	72	58.8	6.48	left	female	no	37	Aspiration Pneumonia	25	0.9	no	no	CEU
S25	42	47	6.44	left	male	yes	8	Hanging	128	1.0	yes	yes	CEU
S26	22	37	6.03	left	male	no	3	Pericarditis	450	1.4	no	no	CEU
S27	38	50	6.02	left	male	no	4	Meningoencephalitis	100	0.4	yes	no	CEU

S28	27	22	6.28	left	male	yes	8	Burning	1200	9.6	no	yes	CEU
S29	48	52.5	6.21	left	female	no	22	Pulmonary Thromboembolism	700	15.4	no	no	CEU
S30	47	41.5	6.52	left	male	yes	21	Multiple Injuries	1400	29.4	no	yes	CEU
Control group 2													
C31	42	30.5	6.45	left	male	no	N/A	Ischaemic Heart Disease	N/A	N/A	N/A	N/A	CEU
C32	52	33.75	6.52	left	male	no	N/A	Ischaemic Heart Disease	N/A	N/A	N/A	N/A	CEU
C33	47	24	5.89	left	female	no	N/A	Pulmonary Embolism	N/A	N/A	N/A	N/A	CEU
C34	75	53	6.01	left	female	no	N/A	Multiple Organ Failure	N/A	N/A	N/A	N/A	CEU
C35	70	59	6.11	left	male	no	N/A	Ischaemic Heart Disease	N/A	N/A	N/A	N/A	CEU
C36	75	69.4	6.19	left	male	no	N/A	Cardiogenic Shock	N/A	N/A	N/A	N/A	CEU
C37	55	30.5	6.69	left	male	no	N/A	Coronary Artery Atherosclerosis	N/A	N/A	N/A	N/A	CEU
C38	66	71.75	6.47	left	male	no	N/A	Coronary Artery Atheroma	N/A	N/A	N/A	N/A	CEU
C39	80	55	6.28	left	female	no	N/A	Ischaemic Heart Disease	N/A	N/A	N/A	N/A	CHB
Major depressive disorder													
MDD1	37	57.75	6.84	left	male	yes	14	Hanging	N/A	N/A	N/A	N/A	CEU
MDD2	51	41	6.71	left	male	yes	15	Hanging	N/A	N/A	N/A	N/A	CEU
MDD3	50	50.5	6.85	left	female	yes	40	Mixed drug toxicity	N/A	N/A	N/A	N/A	CEU
MDD4	77	16.7	6.49	left	female	yes	26	Drug Toxicity	N/A	N/A	N/A	N/A	CEU
MDD5	69	44.5	6.45	left	male	yes	20	Drowning	N/A	N/A	N/A	N/A	CEU
MDD6	79	24	6.32	left	male	yes	17	CO poisoning	N/A	N/A	N/A	N/A	CEU
MDD7	55	47.75	6.6	left	male	yes	3	Hanging	N/A	N/A	N/A	N/A	CEU
MDD8	68	60.75	6.65	left	male	yes	9	Hanging	N/A	N/A	N/A	N/A	CEU
MDD9	87	24.5	6.44	left	female	no	7	Chest Infection	N/A	N/A	N/A	N/A	CEU

MDD10	51	23.5	6.49	left	male	no	25	Drug Toxicity	N/A	N/A	N/A	N/A	CEU
Bipolar disorder													
BP1	38	24	6.42	left	male	yes	3	CO Poisoning	300	0.9	N/A	N/A	CEU
BP2	59	34	6.46	left	male	no	24	Ruptured Aorta	N/A	N/A	N/A	N/A	CEU
BP3	42	25	6.54	left	female	yes	20	Hanging	N/A	N/A	N/A	N/A	CEU
BP4	64	26	6.46	left	female	no	8	Ischaemic Heart Disease	N/A	N/A	N/A	N/A	CEU
BP5	66	17	6.41	left	male	no	12	Food Aspiration	166	2	N/A	N/A	CEU
BP6	79	8.25	6.09	left	male	no	17	Cholecystitis, Cardiomegaly	67.5	14.8	N/A	N/A	CEU
BP7	59	37.5	5.97	left	male	no	8	Ischaemic Heart Disease	N/A	N/A	N/A	N/A	CEU
BP8	61	58	6.44	left	male	no	40	Acute Myocardial Infarction	1500	60	N/A	N/A	CEU
BP9	74	45	6.26	left	female	no	35	Mixed Drug Toxicity	100	3.5	N/A	N/A	CEU
BP10	56	36	6.43	left	female	yes	36	CO poisoning	N/A	N/A	N/A	N/A	CEU

[a]Chlorpromazine equivalents; PMI, post-mortem interval; DOI, duration of illness; BZP, benzodiazepine; CEU, western European ancestry; CHB, Han Chinese ancestry; N/A, not applicable; N/D, not determined.

Supplementary Table 2: Primer sequences

Real-time PCR		
Gene target	Accession no.	Primer sequence
		Human
SNCA	**NM000345**	Forward: CTGCTGCTGAGAAAACCAAA,
		Reverse: CTGCTCCCTCCACTGTCTT
GAPDH	**NM002046**	Forward: TGCACCACCAACTGCTTAGC,
		Reverse: CATGGACTGTGGTCATGAG
PPIA	**NM021130**	Forward: ATGGTCAACCCCACCGTGTTCTTCG,
		Reverse: CGTGTGAAGTCACCACCCTGACACA
SELENBP1	**NM003944**	Forward: TCGCATCTATGTGGTGGAC,
		Reverse: GGCTGGTGTGGAGAAAGG
		Rat
Sdha	**NM130428**	Forward: GTCCATACACCGAATAAGAG,
		Reverse: GCCAGCACCATAGATACC
Map2k5	**NM017246**	Forward: GTAAGAAGTGACGAAGAGATGAAG,
		Reverse: GCTGGTGTGCTGAGATGG
Mapk6	**NM031622**	Forward: GAGAGGCAAGTCCAAGTG,
		Reverse: AAGGCATCAAAGTCAAAGC
Selenbp1	**NM080892**	Forward: AGTGTGGTCCAGGTTATGC,
		Reverse: GCTCGTCCTTCAGGTGTG

SNP analysis	
SNP ID	Primer sequence
rs10788804	Forward: ACGTTGGATGCTCTTCCTGCAATGTTTGCG,
	Reverse: CGTTGGATGTCCGAGGAAGAGCTAGATGG
	Extension: GGTGCAGAGTATAAGGAGG
rs2800953	Forward: ACGTTGGATGTGCCTAGCACAGACATTCAC,
	Reverse: ACGTTGGATGACTTCTGCCTGAACCCTAAC
	Extension: GGGGGGCCTAACTGGCCGTATTTAT

Supplementary Table 3: Association of *SELENBP1* mRNA with potential confounds

		Schizophrenia cohort			Affective disorder cohort
		BA9	BA44	BA8	BA9
Age (years)	r^2	<0.01	<0.01	<0.01	<0.01
	p	0.82	0.62	0.50	0.80
PMI (hours)	r^2	<001	0.03	0.03	0.04
	p	0.97	0.22	0.16	0.33
Brain pH	r^2	0.02	0.13	0.17	0.24
	p	0.34	<0.01	<0.01	<0.01
RIN	r^2	0.03	<0.01	0.02	0.13
	p	0.22	0.69	0.33	0.06
DOI (years)	r^2	<0.01	<0.01	<0.01	<0.01
	p	0.76	0.81	0.92	0.89
Drug dose	r^2	0.01	0.04	<0.01	0.60
(mg)[a]	*p*	0.53	0.84	0.95	0.12
Lifetime exposure (mg)[a]	r^2	0.04	<0.01	0.04	0.07
	p	0.31	0.97	0.26	0.48

[a]Chlorpromazine equivalents; PMI, post-mortem interval (hours); RIN, RNA integrity number; DOI, duration of illness (years).

Supplementary Table 4: Association of SELENBP1 protein with potential confounds

		Schizophrenia cohort		
		BA9	BA44	BA8
Age (years)	r^2	0.06	<0.01	0.08
	p	0.05	0.68	0.03
PMI (hours)	r^2	0.02	0.03	0.03
	p	0.30	0.19	0.26
Brain pH	r^2	0.02	<0.01	0.05
	p	0.30	0.68	0.07
DOI	r^2	0.01	0.03	0.08
	p	0.60	0.35	0.12
Drug dose (mg)[a]	r^2	0.33	0.02	0.01
	p	0.57	0.46	0.56
Lifetime exposure (mg)[a]	r^2	<0.01	0.03	<0.01
	p	0.96	0.33	0.71

[a]Chlorpromazine equivalents; PMI, post-mortem interval; DOI, duration of illness.

Supplementary Table 5: Association of *SELENBP1* mRNA with gene variant at rs10788804

		Schizophrenia vs Control		
		BA9	BA44	BA8
genotype	F	2,50 = 0.32	2,50 = 2.42	2,50 = 0.87
	p	0.73	0.10	0.43
A allele	F	1,50 = 0.08	1,50 = 0.09	1,50 = 0.40
	p	0.78	0.76	0.53
G allele	F	1,52 = 0.51	1,52 = 3.81	1,52 = 1.74
	p	0.48	0.06	0.19

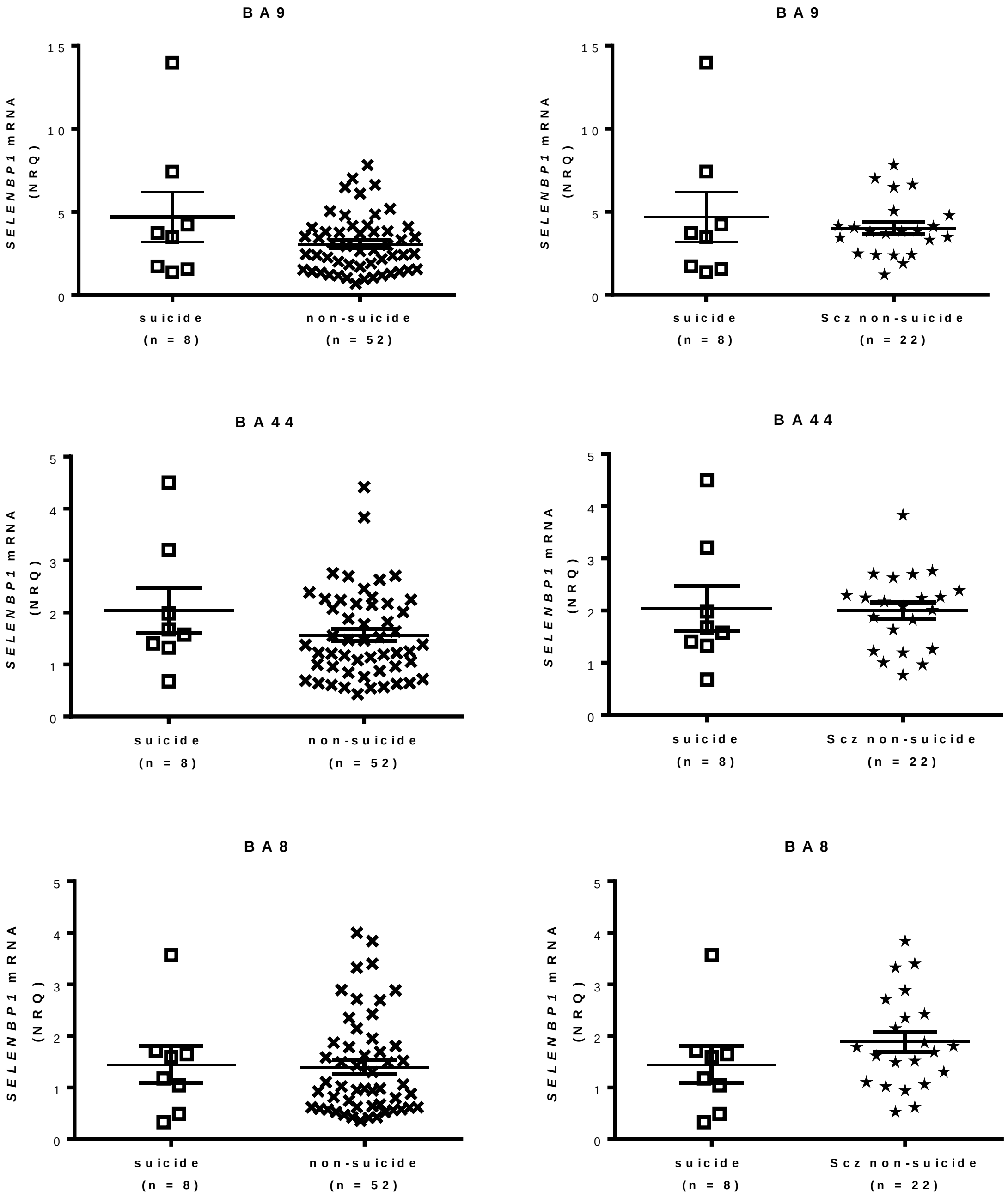

Supplementary Figure 1: *SELENBP1* mRNA expression in BA9 (top), BA44 (middle) and BA8 (bottom) from subjects that died of suicide (□) compared to non-suicide total (×; left) or within schizophrenia (★; right). Bars represent mean ± SEM.

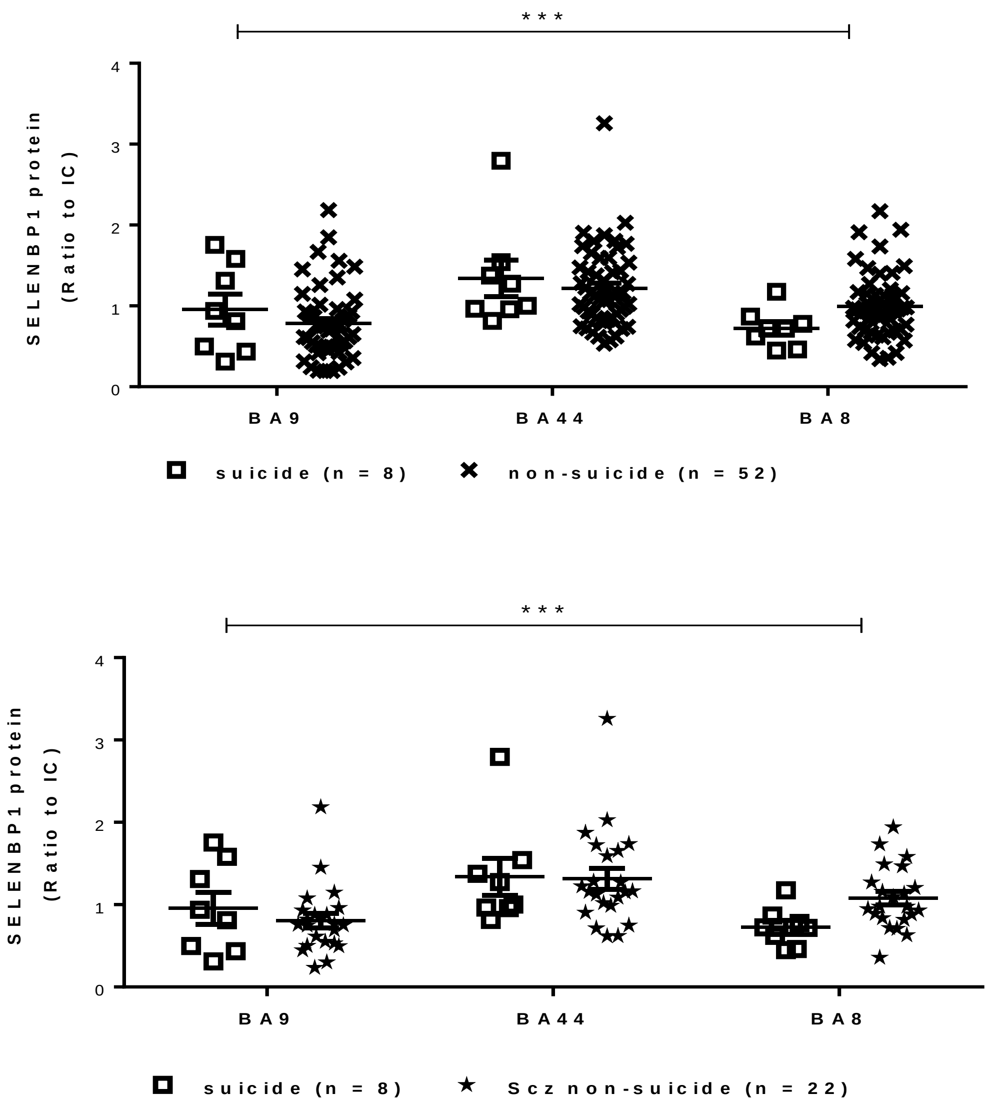

Supplementary Figure 2: *SELENBP1* protein expression in BA9, BA44 and BA8 from subjects that died of suicide (□) compared to non-suicide suicide total (×; top) or within schizophrenia (★; bottom). Bars represent mean ± SEM.
